# SARS-CoV-2 damages cardiomyocyte mitochondria and implicates long COVID-associated cardiovascular manifestations

**DOI:** 10.1016/j.jare.2025.05.013

**Published:** 2025-05-10

**Authors:** Wenliang Che, Shuai Guo, Yanqun Wang, Xiaohua Wan, Bingyu Tan, Hailing Li, Jiasuer Alifu, Mengyun Zhu, Zesong Chen, Peiyao Li, Lei Zhang, Zhaoyong Zhang, Yiliang Wang, Xiaohan Huang, Xinsheng Wang, Jian Zhu, Xijiang Pan, Fa Zhang, Peiyi Wang, Sen-Fang Sui, Jincun Zhao, Yawei Xu, Zheng Liu

**Affiliations:** a Department of Cardiology, Shanghai Tenth People’s Hospital, Tongji University School of Medicine, Shanghai, China; b Cryo-electron Microscopy Center, Southern University of Science and Technology, Shenzhen, China; c School of Life Science, Southern University of Science and Technology, Shenzhen, China; d State Key Laboratory of Respiratory Disease, National Clinical Research Center for Respiratory Disease, Guangzhou Institute of Respiratory Health, The First Affiliated Hospital of Guangzhou Medical University, Guangzhou, China; e School of Medical Technology, Beijing Institute of Technology, Beijing, China; f Shanghai NanoPort, Thermo Fisher Scientific Inc., Shanghai, China; g Guangzhou National Laboratory, Bio-Island, Guangzhou, China

**Keywords:** Long COVID, SARS-COV-2, Endomyocardial biopsy, Mitochondria disorder

## Abstract

**Introduction:**

With the COVID-19 pandemic becoming endemic, vigilance for Long COVID-related cardiovascular issues remains essential, though their specific pathophysiology is largely unexplored.

**Objectives:**

Our study investigates the persistent cardiovascular symptoms observed in individuals long after contracting SARS-CoV-2, a condition commonly referred to as “Long COVID”, which has significantly affected millions globally.

**Methods:**

We meticulously describe the cardiovascular outcomes in five patients, encompassing a range of severe conditions such as sudden cardiac death during exercise, coronary atherosclerotic heart disease, palpitation, chest tightness, and acute myocarditis.

**Results:**

All five patients were diagnosed with myocarditis, confirmed through endomyocardial biopsy and histochemical staining, which identified inflammatory cell infiltration in their heart tissue. Crucially, electron microscopy revealed widespread mitochondrial vacuolations and the presence of myofilament degradation within the cardiomyocytes of these patients. These findings were mirrored in SARS-CoV-2-infected mice, suggesting a potential underlying cellular mechanism for the cardiac effects associated with Long COVID.

**Conclusion:**

Our findings demonstrate a profound impact of SARS-CoV-2 on mitochondrial integrity, shedding light on the cardiovascular implications of Long COVID.

## Highlights

• Our investigation provides histopathological and electron microscopic analyses from endomyocardial biopsies of five Long COVID patients.
• Electron microscopy revealed widespread mitochondrial disorder and the presence of myofilament degradation.
• Similar mitochondria disorganization were found in SARS-CoV-2 infected mice.
• Proteomic profiling indicated that the most significantly changed genes and proteins are related to mitochondrial structure and function.

## Graphical abstract

**Figure:**
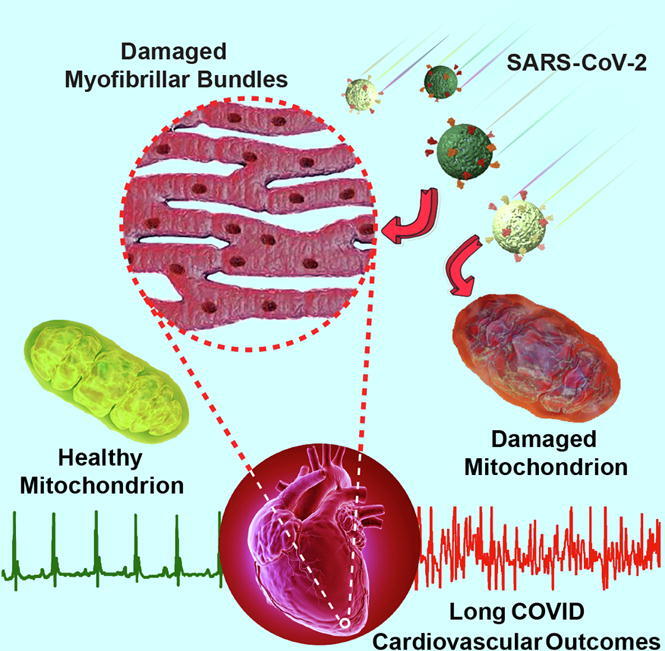

## Introduction

Severe acute respiratory syndrome coronavirus 2 (SARS-CoV-2), the causative agent of the coronavirus disease 2019 (COVID-19) pandemic, emerged in late 2019 and rapidly spread around the world. By the end of September 2024, there have been more than 776 million confirmed cases of COVID-19, with over 7.0 million fatalities worldwide (https://covid19.who.int/). Though primarily affecting the respiratory system, SARS-CoV-2 is also linked to multi-organ dysfunctions, with a notable impact on the cardiovascular system. The virus utilizes the angiotensin-converting enzyme 2 (ACE2) receptor, which is abundantly expressed in cardiomyocytes, to enter human cells, thereby underlining a pathway to cardiac involvement [1,2]. Several autopsy studies have detected SARS-CoV-2 RNA and proteins in cardiomyocytes in the myocardium of deceased COVID-19 patients, indicating direct infection of heart muscle cells during acute phase of COVID-19 [[3], [4], [5]]. Clinical reports have underscored the cardiovascular complications associated with COVID-19, including myocardial injury, arrhythmias, and severe clinical outcomes [[6], [7], [8]].

Beyond the acute phase, a considerable number of recovered patients experience long-term cardiovascular issues, such as cerebrovascular disorders, dysrhythmias, and myocarditis, contributing to the spectrum of “Post-acute COVID-19 Syndrome” or “Long COVID” [9,10]. Long COVID is broadly defined as signs, symptoms, and conditions that continue or develop after initial COVID-19 or SARS-CoV-2 infection. The signs, symptoms, and conditions persist for four weeks or more after the initial phase of infection. This working definition was developed by the U.S. Department of Health and Human Services in collaboration with CDC, NIH, and medical societies (https://www.covid.gov/longcovid/). A recent longitudinal cohort study has reported that approximately one in eight patients, or 12.7 %, have persistent symptoms up to 150 days attributed to COVID-19, with chest pain among the most common Long COVID symptoms [11]. A latest research demonstrates the risk of major adverse cardiac events (MACE) was elevated in COVID-19 cases, including myocardial infarction, stroke, and mortality [12], and a scientific statement from the American Heart Association also advises monitoring cardiac arrhythmias in COVID-19 patients and those in recovery [13]. However, the specific molecular mechanisms and signaling pathways underlying the Long COVID-associated cardiovascular outcomes remain unclear.

With the pandemic transitioning to an endemic phase, continued vigilance for Long COVID-related cardiovascular complications remains essential, especially given the largely unexplored pathophysiology. Recent studies also suggest that post-COVID cardiovascular monitoring strategies may need to differ based on patients’ vaccination status [14,15]. Our investigation provides histopathological and electron microscopic analyses from endomyocardial biopsies of five patients who experienced various cardiovascular outcomes, such as acute myocarditis and myocardial fibrosis, within one to three months post-COVID-19 recovery. Findings include inflammatory cell infiltration, myofilament degradation, and notably, extensive mitochondrial damage within cardiomyocytes. Mitochondria are vital cellular organelle function for most essential cellular processes, such as ATP synthesis, fatty acid oxidation, metabolic activity, calcium storage, and apoptosis. Damage to mitochondrial structure leads to impaired mitochondrial function, which directly compromises human health. In cardiomyocytes, structural damage to mitochondria disrupts ATP synthesis, resulting in insufficient energy supply required for proper cardiac contraction. This energy deficit can trigger a range of cardiac disorders, including arrhythmias, palpitations, fatigue, and chest pain. These observations suggest a profound impact of COVID-19 on mitochondrial integrity, potentially elucidating a cellular mechanism behind the cardiovascular sequelae of Long COVID.

## Methods

### Clinical study

All Clinical studies were approved by the Human Ethics Committee of Shanghai Tenth People’s Hospital, Tongji University School of Medicine (Approval Number: 23KT45). Written informed consent was obtained from the patient. The studies were performed in accordance with the Declaration of Helsinki.

### Endomyocardial biopsy

An endomyocardial biopsy was performed to determine the cause of the patient's sudden cardiac arrest. Using a disposable Cordis bioptome guided by fluoroscopy through the right femoral vein, six tissue fragments were obtained from the right ventricular septum. For immunohistochemical, Masson's trichrome staining (a three-color staining procedure used in histology for selectively stain collagen, collagen fibers, fibrin, muscles, and erythrocytes), and hematoxylin-eosin (HE) staining (a principal tissue stain used in histology to illustrate nuclear and cytoplasmic components, to identify different types of cells and tissues), tissue samples were fixed in formalin and embedded in paraffin, 5 μm thick sections were examined under light microscopy.

### Transmission electron microscopy

Myocardial tissue was pre-fixed with 1 % glutaraldehyde followed by 1 % osmium tetroxide fixation. The tissue was dehydrated step by step using a gradient ethanol solution, followed by gradient infiltration with a mixture of acetone and Epon812 resin and resin-embedded polymerization. The resin block was cut into 70 nm ultrathin sections, double-stained with uranyl acetate and lead citrate, and the specimen was observed under transmission electron microscopy (Talos L120C G2, Thermo Fisher Scientific) operating at 120 kV. Images were recorded with a Ceta 4 K × 4 K CMOS camera.

### Focused ion beam-scanning electron microscopy (FIB-SEM)

The resin block was observed under a dual beam scanning electron microscope (Aquilos Cryo-FIB, Thermo Fisher Scientific). Each serial face was imaged using T1 (in-lens detector) backscatter mode, with a 2.0 keV acceleration voltage and a current of 0.1 nA. The pixel size of X-Y panels was 5 nm, and image dimension varied for each dataset (about 10–30 μm). The resin block was sequentially milled by a gallium ion beam, with a Z-axis height of 10 nm for each milling, and number of images varied for each dataset (about 800–1800). Images were aligned, cropped, and binned to form an image stack with 10 nm isotropic resolution in X-Y-Z dimension for further segmentation and analysis.

### Viruses and mice

The BALB/c mice were purchased from Jinan Pengyue Experimental Animal Breeding Co. LTD. The SARS-CoV-2 variants Omicron BQ.1 and BA.5.2 were isolated from COVID-19 patients in Guangdong, China. Experiments related to authentic SARS-CoV-2 were conducted in Guangzhou Customs District Technology Center BSL-3 Laboratory.

### Animal experiments

All animal studies were performed following guidelines from the Directive 2010/63/EU of the European Parliament on the protection of animals used for scientific purposes. All protocols were approved by the Institutional Animal Care and Use Committee (IACUC) of The First Affiliated Hospital of Guangzhou Medical University (Approval Number: 20230451). 6–8 weeks old mice were challenged with 1 × 10^5^ FFU SARS-CoV-2 (BQ.1 and BA.5.2). Mice were challenged with phosphate-buffered saline as controls. To investigate the ultrastructure of cardiomyocytes, hearts were collected seven days after infection. Mice were humanely anesthetized by administration of ketamine (80 mg/kg) and xylazine (5 mg/kg). Anesthesia was confirmed by no obvious reflex response to hind feet pinching. After deep anesthesia, animals were euthanized by cervical dislocation. Hearts were excised instantly and myocardial tissue from mice was prepared using the same protocol as endomyocardial biopsy tissue.

### Proteome analysis

4D label-free quantitative proteomics analysis was utilized to screen proteins differentially expressed in infected mouse heart tissue. Briefly, mouse heart tissue was ground individually in liquid nitrogen and lysed with SDT lysis buffer, and protein concentration was determined by BCA method. Protein samples were hydrolyzed by trypsin, and desalination was conducted on the C18 desalting column, and peptides were collected and lyophilized. The peptides were dissolved and fractionated using a C18 column using a Rigol L3000 HPLC system. The eluates were separated and analyzed with a nano UPLC (EVOSEP One, Denmark) coupled to a timsTOF Pro2 mass spectrometry (Bruker, Germany) with a nano-electrospray ion source. The mass spectrometer adopts DDA PaSEF mode for data acquisition, and the full scan MS survey spectra range was set from 100 to 1700 *m*/*z*. The raw files were processed using SpectroMine software (Ver. 4.2.230428.52329, Biognosys AG, Switzerland), MS spectra lists were searched against their species-level UniProt FASTA databases [16]. Peptide identification was performed with an initial precursor mass deviation of up to 20 ppm and a fragment mass deviation of 20 ppm. Gene Ontology (GO) and InterPro (IPR) functional analysis were conducted using the InterProScan program against the non-redundant protein database [17], and the databases of Clusters of Orthologous Groups (COG) and Kyoto Encyclopedia of Genes and Genomes (KEGG) were used to analyze the protein family and pathway [18,19].

## Results

### Ultrastructural disorder in cardiomyocytes in a patient suffer sudden cardiac arrest

**Fig. 1:**
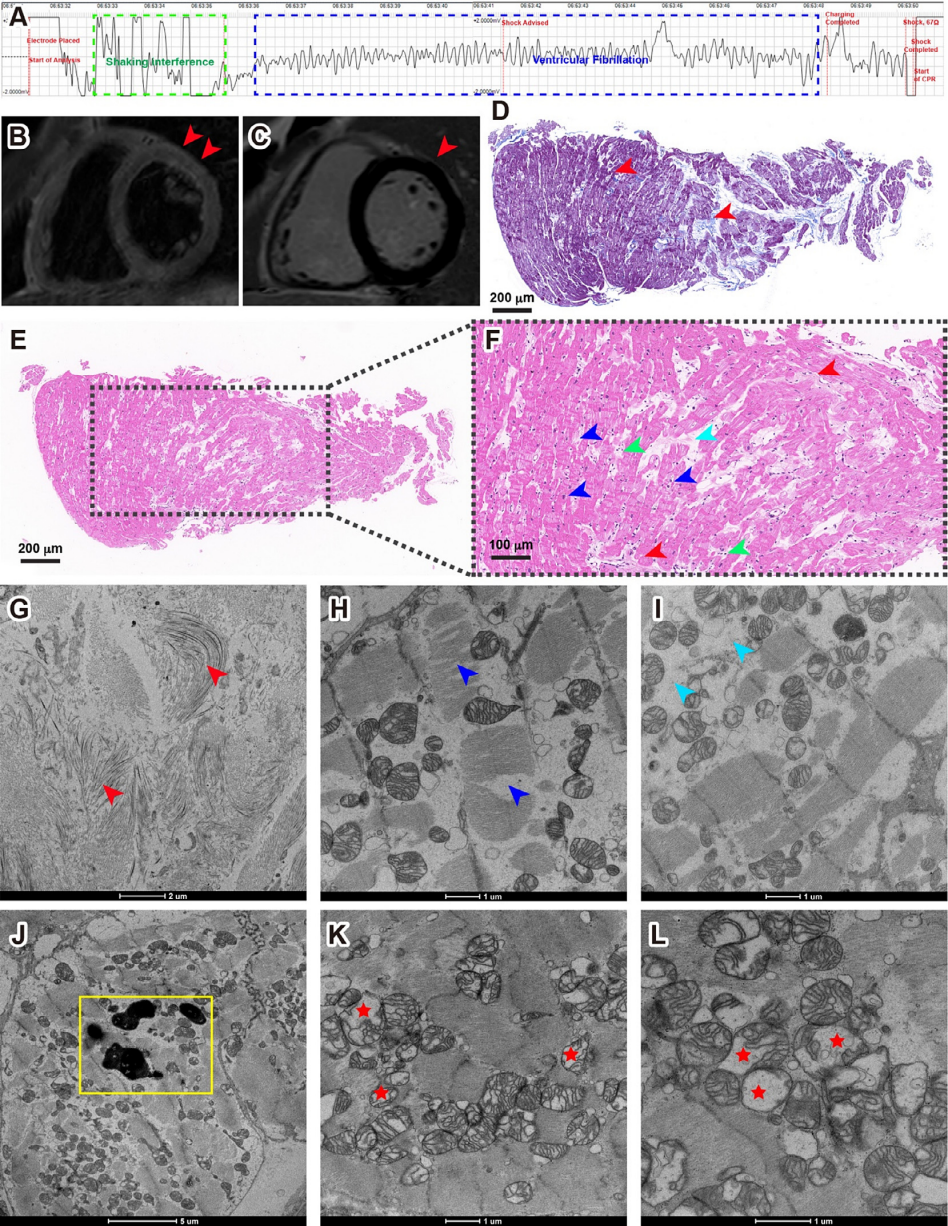
**Spectrum of myocarditis in COVID-19 Patient #1. (A)** ECG recordings retrieved from the AED, shows coarse ventricular fibrillation before the shock (blue frame). **(B)** CMR imaging of the patient, both T2-weighted sequences (T2WI) and short inversion time inversion recovery sequences (STIR) showed no myocardial edema in the mid short-axis slices. **(C)** A late enhancement was not observed in late gadolinium enhancement (LGE) sequences of the mid short-axis slices in the myocardium. Red arrowheads indicated suspected myocardial edema under the epicardium of the anterior wall (basal part), without significant delayed enhancement. **(D)** Masson's trichrome staining displayed interstitial collagen fiber deposits (red arrowheads). **(E)** HE analysis shows an abnormal structure in the myocardium. **(F)** Zoomed view, shows interstitial collagen fiber accumulation (red arrowheads), loss of integrity of myofibrillar bundles (blue arrowheads), and edema and necrosis of myofibrillar bundles (cyan arrowhead); Myofibrillar interstitial infiltration with inflammatory cells is also observed (green arrowheads). **(G)** Electron micrographs of the biopsy sample from the patient, showing local myofibrillar fibrosis and proliferation of fibroblasts (red arrowheads); **(H)** Loss of integrity of myofibrillar bundles (blue arrowheads); **(I)** Necrosis of myofibrillar bundles (cyan arrowheads); **(J)** Lipofuscin granules in the cardiomyocytes (yellow frames); **(K)** and **(L)** A significant amount of vacuolations in the mitochondria (red stars). (For interpretation of the references to color in this figure legend, the reader is referred to the web version of this article.)

A 30-year-old male suffered a syncope during exercise, leading to lost consciousness, cessation of breathing, and absence of carotid pulse. Immediate intervention with two cycles of cardiopulmonary resuscitation (CPR) and automated external defibrillator (AED) deployment successfully restored his heartbeat and breathing, although he remained unconscious. Upon regaining consciousness en route to Shanghai Tenth People’s Hospital, the patient had limited recollection of the event. Initial assessment in the coronary care unit (CCU) for further diagnosis. The electrocardiogram (ECG) at the time of the AED discharge indicated ventricular fibrillation, prompted further investigation (Fig. S1 and Fig. 1**A**).

The patient has recently recovered from COVID-19, 37 days prior to the incident, was notable in the absence of any chronic health conditions or familial cardiovascular disease history. Comprehensive laboratory assessments revealed generally normal results, with specific abnormalities including elevated cardiac biomarkers (Troponin T, creatine kinase-MB, and myoglobin) and abnormal neutrophil counts indicative of myocardial injury and inflammation, respectively (Table S1). All pathogen tests, including SARS-CoV-2 and Coxsackie virus, were negative. Additional investigations resulted in no signs of rheumatic heart disease, autoimmune disease, and tumors that may cause myocardial injury. We considered possible viral myocarditis (VMC), however, only suspicious myocardial edema in the anterior subepicardial with no significant delayed enhancement in cardiac magnetic resonance (CMR, Fig. 1B and C). Coronary angiography (CAG) confirmed unobstructed coronary arteries. Further diagnostic tools, including ECG, echocardiography (Echo), 24-h dynamic electrocardiogram, endocardial electrophysiological examination, and genetic analysis, revealed no abnormalities.

Endomyocardial biopsy remains the gold standard mode of investigation for diagnosing many cardiac conditions, including suspected myocarditis, heart failure of unknown etiology, cardiomyopathy, arrhythmia, heart transplant rejection and secondary involvement by systemic diseases [20]. To further clarify the pathogeny of the patient, an endomyocardial biopsy was performed. The biopsy sample underwent immunohistochemical analysis to characterize inflammatory cell infiltration by using the following antibodies: CD3, CD4, CD8, CD9, CD20, and CD68. Only CD68 showed weekly positive, which indicated a minimal presence of macrophages (Table S1). A Masson's trichrome staining highlighted interstitial collagen fiber deposition, suggesting fibrosis (Fig. 1D), which were also confirmed as fibrosis in HE staining and under electron microscopy (Fig. 1E, F, G, and Fig. S2A). Both HE staining and electron microscopy revealed loss of myofibrillar bundles, interstitial oedema and necrosis (Fig. 1F, H, I, Fig. S2B, and C), which indicated myocytes degeneration. Notably, electron microscopy examination revealed lipofuscin granules accumulation in myocardial cells (Figs. 1J and S2D), the same kind of lipofuscin pigments was previously observed in sudden cardiac death [21]. Interestingly, we observed disordered myocardial ultrastructure in the biopsy tissue, especially in the mitochondria: a significant number of mitochondria were swollen and vacuolated, with distorted and broken cristae (Fig. 1K, L, and Fig. S2E). Swollen and degenerating mitochondria were previously observed in autoimmune myocarditis in rat models [22,23]. Unsurprisingly, we did not observe any SARS-CoV-2 virus in the endomyocardial biopsy sample, since the patient has test negative for almost a month. The immunohistochemical analysis, Masson's trichrome staining, HE staining, and electron microscopic analysis aid in the diagnosis of myocarditis [24]. Throughout his hospitalization, Patient #1 received guideline-based care, including cardiac monitoring, oxygen therapy, and antiarrhythmic medications, leading to a stable discharge after ten days.

### Ultrastructural disorder in cardiomyocytes in a patient suffer chest tightness, palpitation and fatigue

Patient #2 is a 68-year-old male admitted to Gansu Provincial People's Hospital with Long COVID symptoms including chest tightness, palpitations, and fatigue persisting for three weeks since recovery from COVID-19, he tested SARS-CoV-2 positive 31 days prior to hospital admission. For further evaluation and treatment, the patient was transferred to the Department of Cardiology at Shanghai Tenth People’s Hospital. His medical history was notable for hypertension and coronary atherosclerotic heart disease. Initial diagnostics revealed abnormal Q waves and ST segment depression in the III and avF leads on the ECG, and an Echo indicated impaired diastolic heart function. Laboratory tests showed mildly elevated cardiac troponin T (cTnT) levels. CAG found no evidence of coronary stenosis, but CMR imaging displayed delayed enhancement in the anterior septal muscle layer of the left ventricle's basal and middle levels, suggestive of myocardial fibrosis.

**Fig. 2:**
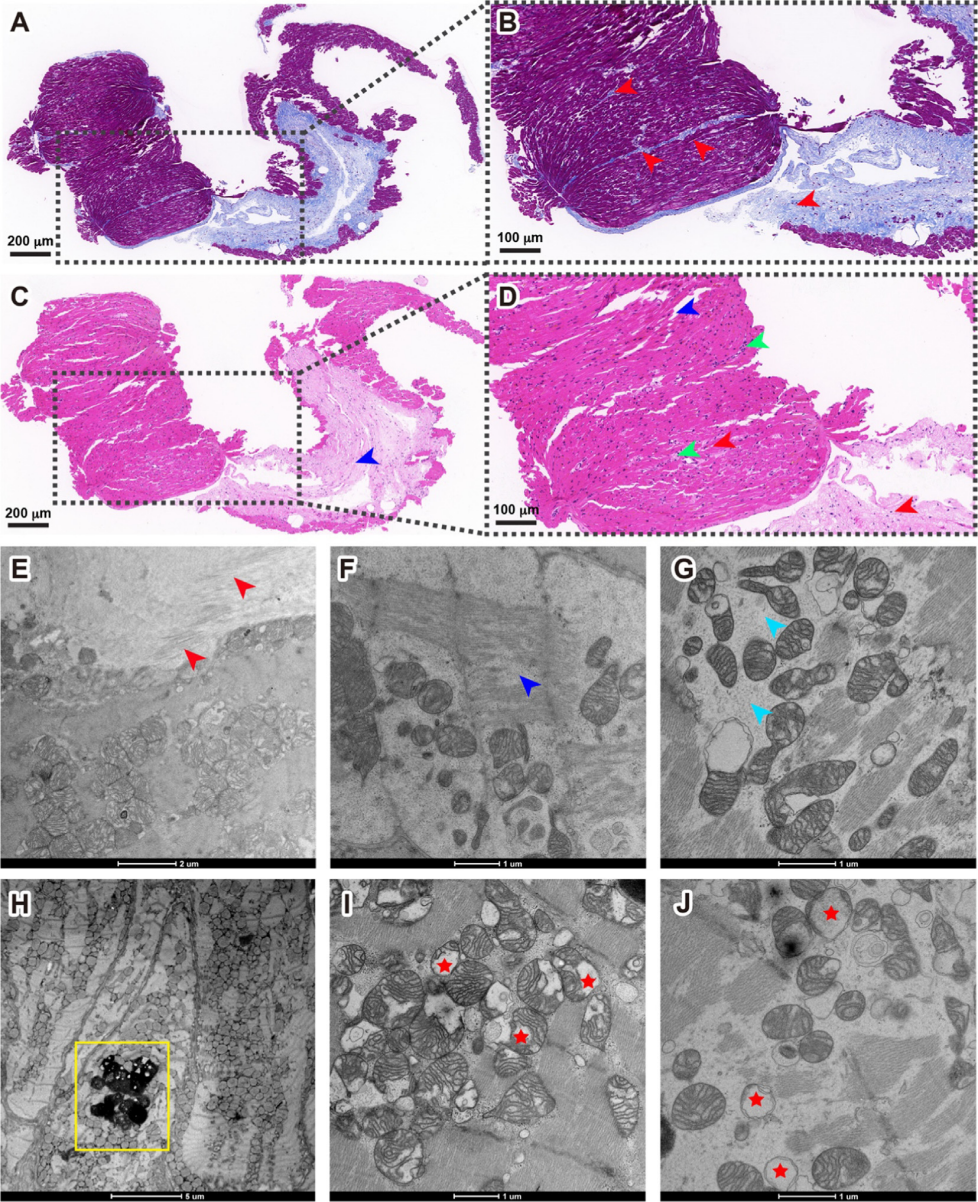
**Spectrum of myocarditis in COVID-19 Patient #2. (A)** Masson's trichrome staining. **(B)** Zoomed view displayed interstitial collagen fiber deposits (red arrowheads). **(C)** HE analysis shows an abnormal structure in the myocardium; **(D)** Zoomed view, shows interstitial collagen fiber accumulation (red arrowheads), loss of integrity of myofibrillar bundles (blue arrowheads), and myofibrillar interstitial infiltration with inflammatory cells is also observed (green arrowheads). **(E)** Electron micrographs of the biopsy sample from the patient, showing local myofibrillar fibrosis and proliferation of fibroblasts (red arrowheads); **(F)** Loss of integrity of myofibrillar bundles (blue arrowheads); **(G)** Necrosis of myofibrillar bundles (cyan arrowheads); **(H)** Lipofuscin granules in the cardiomyocytes (yellow frames); **(I)** and **(J)** A significant amount of vacuolations in the mitochondria (red stars). (For interpretation of the references to color in this figure legend, the reader is referred to the web version of this article.)

An endomyocardial biopsy performed 38 days post COVID-19 infection, subsequent analysis revealed significant findings. Masson's trichrome staining highlighted interstitial collagen fiber deposition (Fig. 2A and B). HE staining demonstrated interstitial collagen accumulation, disrupted myofibrillar bundle integrity, and interstitial infiltration by inflammatory cells (Fig. 2C and D). Immunohistochemical analysis showed a lack of reactivity for CD3, CD4, CD8, CD9, and CD20, with only a weak positive signal for interstitial CD68 (Table 1). Electron microscopy further detailed local myofibrillar fibrosis, fibroblast proliferation, muscle bundle edema, loss of myofibrillar integrity, presence of lipofuscin granules in cardiomyocytes, and significant mitochondrial vacuolation (refers loss of cristae structure and formation of vacuoles or vacuole-like mitochondria, Fig. 2E–J), mirroring the pathological changes observed in Patient #1 and reinforcing the diagnosis of myocarditis.

**Table 1:** Summary data for 5 patients.

Throughout his hospitalization, the patient underwent rigorous cardiac monitoring and was administered a comprehensive treatment regimen that included antiplatelet, antihypertensive, lipid-regulating, and myocardial remodeling-improving medications as secondary prevention measures. Following a stable 10-day hospitalization without any MACE, he was discharged, marking a critical step towards recovery.

### Ultrastructural disorder in cardiomyocytes in three patients diagnosed with myocarditis

Patient #3 is a 44-year-old male who recovered from COVID-19 two months prior to admission. This patient does not have any chronic diseases but presented with symptoms of fever and chest tightness. Pathogenetic tests, including SARS-CoV-2 and coxsackievirus, were shown to be negative. ECG suggested sinus tachycardia, elevated ST segments in leads II, III, avF, V5, and V6, as well as abnormal Q waves in III and avF leads. Cardiac enzyme markers, including cTnT, myoglobin, creatine kinase isoenzyme MB (CK-MB), and N-terminal pro-brain natriuretic peptide (NT-pro BNP), were all significantly elevated, indicating myocardial injury. CMR confirmed acute myocarditis, primarily affecting the left ventricular free wall and the proximal region of the inferior wall. A small amount of pericardial effusion and impaired left ventricular systolic function were also observed. Other imaging tests, including chest CT, Echo, and CAG, did not reveal significant abnormalities.

Patient #4 is a 70-year-old male who recovered from COVID-19 two and a half months before being admitted to our hospital. He does not have any chronic diseases but presented with symptoms of chest tightness and dyspnea. ECG revealed sinus rhythm, frequent ventricular premature beats, frequent atrial premature beats, atrial fibrillation, and ST-segment depression. Cardiac enzyme NT-pro BNP levels were elevated. CMR indicated myocardial edema in the left ventricular septum, anterior wall, adjacent anterior septum, and free wall, suggesting myocarditis. Additional imaging tests revealed no significant abnormalities.

Patient #5 is a 33-year-old male who recovered from COVID-19 three months prior to admission. He experienced chest pain, along with elevated cardiac enzyme markers and an increased C-reactive protein (CRP) level. Imaging tests, including ECG, CMR, Echo, chest CT, and CAG, did not indicate significant changes, suggesting a subclinical presentation of myocarditis.

**Fig. 3:**
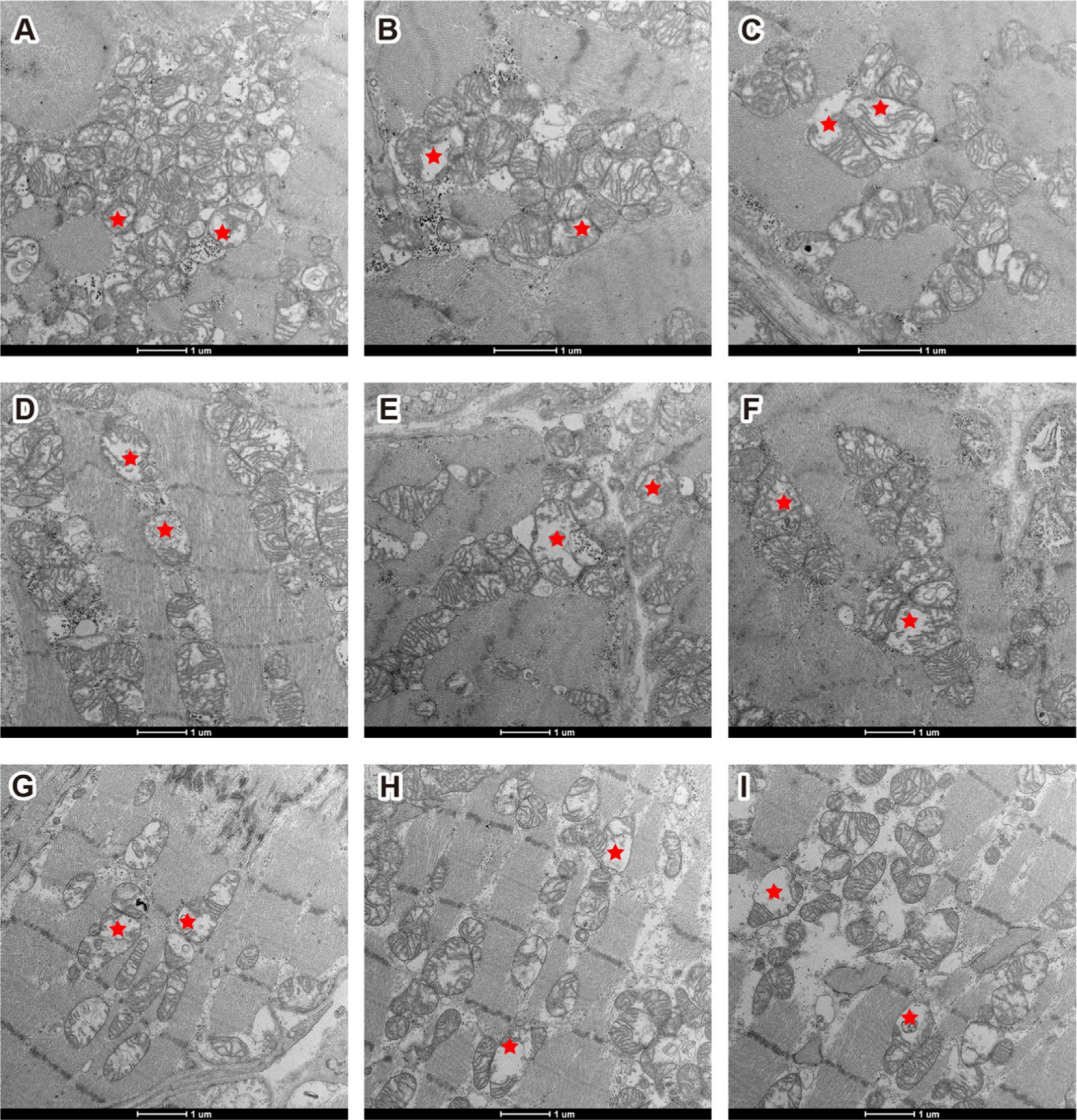
**Mitochondria disorders in COVID-19 patient #3, #4, and #5. (A)**, **(B)**, and **(C)** patient #3. **(D)**, **(E)**, and **(F)** patient #4. **(G)**, **(H)**, and **(I)**, patient #5.

All three patients were diagnosed with myocarditis based on clinical presentation, elevated cardiac markers, and CMR findings. Endomyocardial biopsies conducted at various intervals post-COVID-19 infection revealed interstitial collagen deposits, lipofuscin granules accumulation, loss of integrity and necrosis of myofibrillar bundles, and notably, mitochondrial ultrastructural disorders similar to patients #1 and #2, reinforcing the diagnosis of myocarditis (Fig. 3 and Fig. S3).

### Qualitative and quantitative analysis of mitochondria disorder in 2D images and 3D volumes

**Fig. 4:**
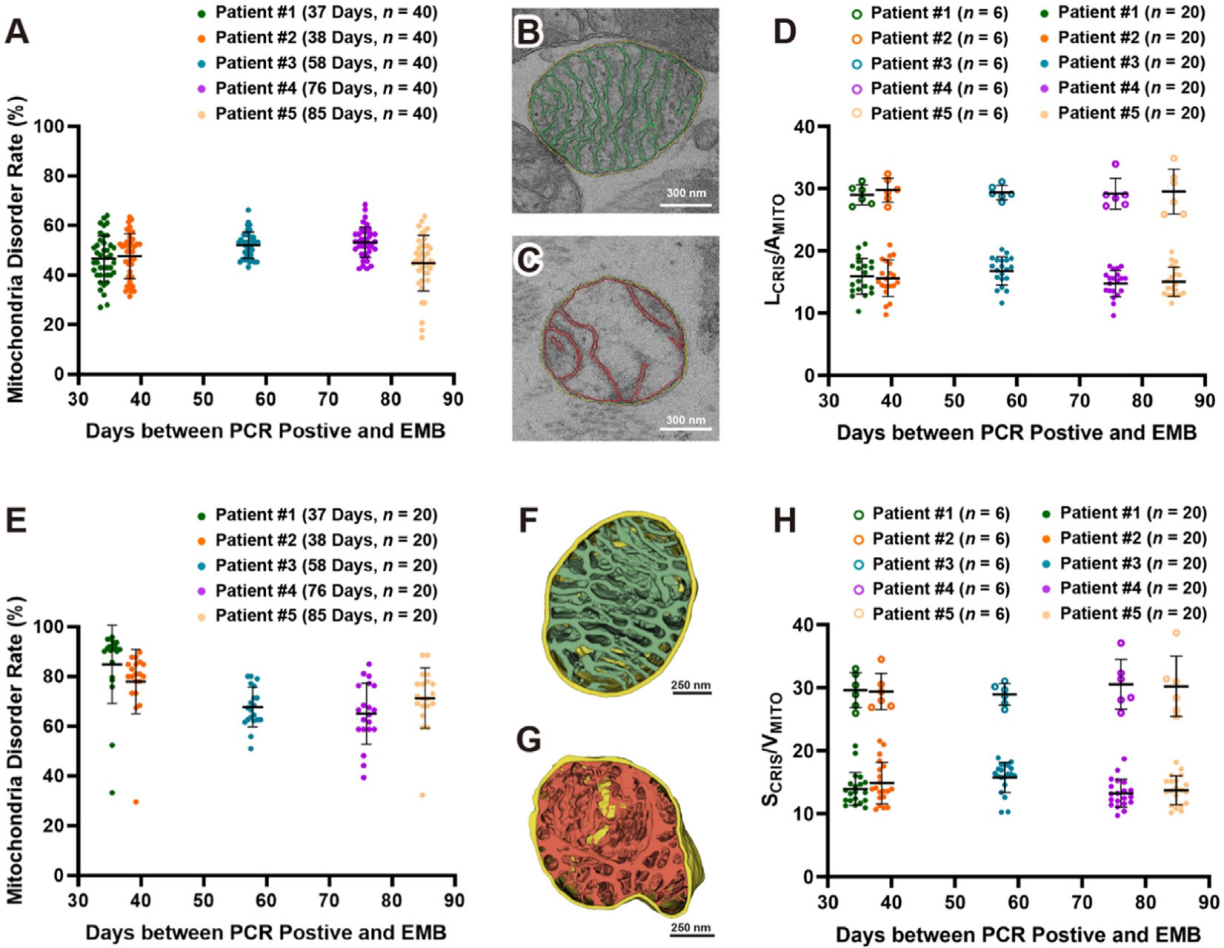
**Qualitative and quantitative analysis of mitochondria disorder in 2D images and 3D volumes. (A)** Analysis of mitochondria disorder rate in 40 cardiomyocytes from each patient, each point represents an averaged percentile of disordered mitochondria from total 80 to 100 mitochondria in each cardiomyocyte. **(B)** and **(C)** 2D segmentation of outer membrane, inner membrane, and cristae in a healthy and disordered mitochondrion, respectively. **(D)** Analysis of mitochondria disorder by comparison the ratio of entire cristae length to the area of the mitochondrial cross section (L_CRIS_/A_MITO_). Healthy mitochondria like the one shown in Panel B have an averaged ratio around 29 μm^−1^ (open circles in top portion of Panel D). In contrast, disordered mitochondria like the one shown in Panel C have a lower ratio (filled circles in lower portion of Panel D). **(E)** Analysis of mitochondria disorder rate in 20 tissue blocks from each patient, each point represents an averaged percentile of disordered mitochondria from total 80 to 100 mitochondria in each block. **(F)** and **(G)** 3D segmentation of outer membrane, inner membrane, and cristae in a healthy and disordered mitochondrion, respectively. **(H)** Analysis of mitochondria disorder by comparison the ratio of surface area of the entire cristae contained within a mitochondrial volume (S_CRIS_/V_MITO_). Healthy mitochondria like the one shown in Panel F have an averaged ratio around 27 μm^−1^ (open circles in top portion of Panel H), disordered mitochondria like the one shown in Panel G have lower ratios (filled circles in lower portion of Panel H).

We conducted a qualitative analysis of mitochondria disorder rate in the myocardial biopsy samples obtained from five patients. Forty cardiomyocytes from each patient were randomly picked, with approximately 80–100 mitochondria in each cardiomyocyte were selected for analysis. We classified them into two groups: healthy or disordered mitochondria (termed as swollen and vacuolated mitochondria, and cristae distorted and broken), and the percentage of the disordered mitochondria rate was calculated for each cardiomyocyte. As shown in Fig. 4**A**, the proportions of disordered mitochondria relative to the total number of mitochondria are approximately 40–60 % for all five patients. This suggests that there is no significant difference in the extent of mitochondrial damage between what we speculated as longer recovery periods (e.g., 76 days and 85 days) and shorter recovery periods (e.g., 37 days and 38 days). It should be noted that the results in Fig. 4**A** did not account for various influencing factors such as patient age, pre-existing health conditions before contracting COVID-19, and the presence of any underlying diseases.

Next, we performed segmentation of the outer mitochondrial membrane (OMM), inner mitochondrial membrane (IMM), and cristae in 2D electron microscope images. Fig. 4**B and C** display segmentation marks for a healthy mitochondrion and a disordered mitochondrion, respectively. We measured the length of the entire cristae (the inner boundary membrane plus the cristae membranes) and calculated the ratio of entire cristae length to the area of the mitochondrial cross section (L_CRIS_/A_MITO_), termed “crista density”, measured in 2D images [25]. This ratio quantifies the degree of damage to the mitochondrial cristae structure. As shown in Fig. 4**D**, the more severe the structural damage to the mitochondria, the more pronounced the cristae loss, resulting in a diminished length of the cristae membrane and a reduced ratio of L_CRIS_/A_MITO_. Our data showed that this ratio in healthy human mitochondria is around 29 μm^−1^, as depicted by open circles in top portion in Fig. 4**D**. In contrast, the ratio dramatically decreased in the disordered mitochondria, and in the worst-case scenario, almost cristae membranes had vanished in the vacuolar mitochondria, and the ratio drops close to 10 μm^−1^ (filled circles in lower portion in Fig. 4**D**). Previous studies have reported that L_CRIS_/A_MITO_ ratios varying from 34, 37, to 41 μm^−1^ in rat myocardial cells [[25], [26], [27]], which are slightly higher than those we measured in human cardiomyocytes.

We further analysis the mitochondria disorder in 3D volumes by focused ion beam-scanning electron microscopy (FIB-SEM) [28], one of the 3D volume electron microscopy techniques that is promised to have an outsized impact on science in the coming years [29,30]. As shown in Movie S1, a tissue fragment from Patient #1 was analyzed by FIB-SEM, a gallium FIB was used to shave 10 nm thin section from the tissue fragment followed by SEM imaging (pixel size 5 nm, in X-Y panel), 930 serial images were binned (convert X-Y pixel size to 10 nm to make 10 nm isotropic resolution in X-Y-Z direction), aligned, cropped (remove misaligned edges), and stacked to form a 3D volume measuring 16 μm × 12 μm × 9.3 μm in X-Y-Z dimensions.

Next, OMM, IMM, and cristae membrane of each mitochondrion were segmented using a deep learning-based MitoStructSeg platform [31], which employs an Adaptive Multi-Domain Mitochondrial Segmentation (AMM-Seg) model specifically developed to accurately segment OMM, IMM, and cristae membranes of mitochondria. As shown in Movie S2, a volume block was selected from a FIB-SEM raw dataset (4 μm × 4 μm, 40 serial images) and displayed in two-channel: raw images and AI-based segmentation images. The raw image is initially submitted to AMM-Seg, where it undergoes rigorous data preprocessing to yield an augmented image and a textured image. Subsequently, the encoder precisely extracts the distinctive features from both the augmented and textured images. The adaptive fusion module then ingeniously integrates these two feature sets to create an enhanced and comprehensive feature representation. Finally, the decoder utilizes the enhanced representation to generate the mitochondrial segmentation results, including OMM, IMM and cristae membranes.

Movie S3 displayed a tissue block that enriched with mitochondria, measuring 4 μm × 4 μm × 9 μm, with all mitochondria were automatically segmented in the block. We classified them into two groups: healthy (shown in green, a selection criterion was that no cristae damage was observed in any section of the 3D volume) and disordered (shown in red). The percentage of the disordered mitochondria rate was calculated. Twenty blocks from each of the five patients were analyzed, with approximately 80–100 mitochondria in each block were selected for analysis. Fig. 4**E** indicated that the proportion of disordered mitochondria rate is approximately 60–90 % for all five patients, more severe than those analyzed in 2D images (Fig. 4**A**). This indicated that mitochondria disorder rates measured in 3D volume are more accurate than those measured in 2D images.

Fig. 4**F and G** illustrated 3D visualization of OMM, IMM, and cristae in a healthy mitochondrion and a disordered mitochondrion, respectively. We quantified crista density in 3D volumes by measure the surface area of the entire cristae contained within a mitochondrial volume (S_CRIS_/V_MITO_). Fig. 4**H** indicated that ratios in healthy human mitochondria are around 28 μm^−1^ (depicted as open circles in top portion in Fig. 4**H**). In contrast, the ratio decreased in the disordered mitochondria (filled circles in lower portion in Fig. 4**H**). The crista density ratios quantified in 3D volumes as S_CRIS_/V_MITO_ are consistent with L_CRIS_/A_MITO_ ratios quantified in 2D images (Fig. 4**D**).

Traditionally, 50–100 nm thin sections of tissue or cell are imaged with conventional transmission electron microscopy, which provides ultrastructural details in 2D (2–10 nm resolution) but often lacks critical spatial information. FIB-SEM has emerged as a powerful tool in cell biology, offing high voxel resolution (5–10 nm), particularly in depth (Z) direction, enabling detailed 3D ultrastructural analysis of tissues and cells. In this study, analysis of cristae spatial organization and mitochondria disorder rates in 3D reconstructions revealed a more severe damage (Fig. 4**A** vs **E**). Although the crista density ratios quantified in 3D volume (S_CRIS_/V_MITO_) were consistent with those measured in 2D images (L_CRIS_/A_MITO_, Fig. 4**D** vs **H**), the 3D analysis provides complementary and reinforcing evidence for the 2D observations.

### Similar mitochondria disorganization found in SARS-CoV-2 Omicron infected mice

We hypothesized the ultrastructural disorders observed in the cardiomyocytes of patients were caused by SARS-CoV-2 infection. Unfortunately, due to several objective limitations, we were unable to include an uninfected control group in the study. All five human myocardial biopsy samples used in our analysis were collected between February and March 2023, during the first major wave of COVID-19 infections in China. These patients were uniquely valuable in that they had detailed nucleic acid testing histories, allowing us to determine the precise timing of infection and recovery. However, with the widespread transmission of SARS-CoV-2 during early 2023 and subsequent waves, it has since become nearly impossible to identify individuals who have never been infected. This has significantly hampered our efforts to recruit uninfected individuals or obtain myocardial tissue from such cases for use as strict healthy controls.

Due to limited resource for endomyocardial biopsy samples and the lack of healthy controls, we investigated these ultrastructural disorders using mice infected with SARS-CoV-2. According to a recent assessment of the diversity of Omicron sublineages and the epidemiologic features of the autumn/winter 2022 COVID-19 wave in Shanghai, BA.5.2 and BQ.1 are the two dominating sublineages [32]. In our animal study, BALB/c mice were challenged with Omicron BA.5.2 and BQ.1 sublineages, heart tissues were collected 7 days post infection (dpi, convalescence stage for mouse infection).

**Fig. 5:**
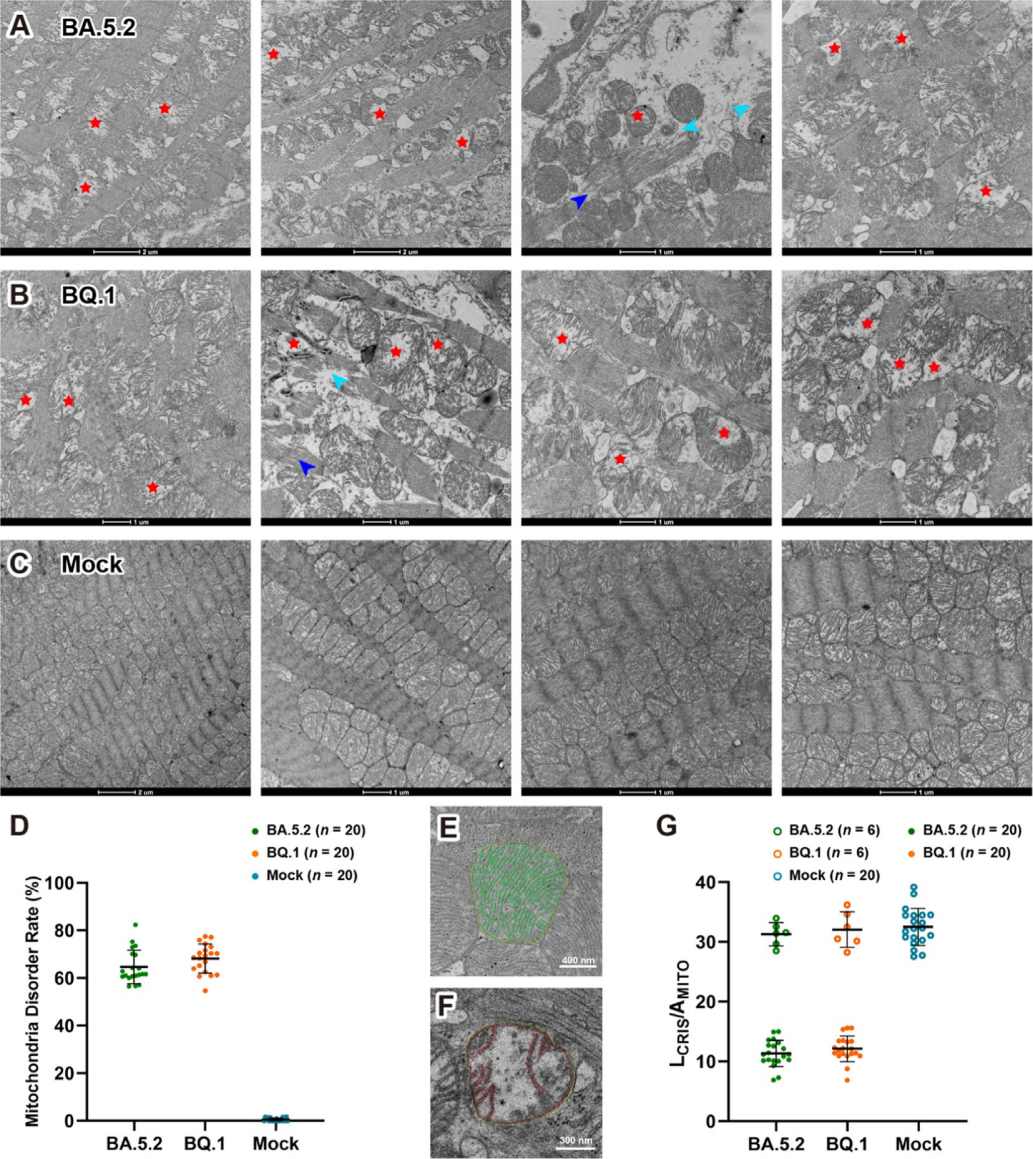
**Mitochondria disorganization in SARS-CoV-2 infected mice. (A)** and **(B)** Electron micrographs of heart tissue from BA.5.2 and BQ.1 infected mice, showing similar disorder (red stars) in the mitochondria and myofibrillar bundles (blue and cyan arrowheads), as observed in the biopsy tissue from the patient. **(C)** Electron micrographs of heart tissue from control group, showing normal mitochondria ultrastructure. **(D)** Analysis of mitochondria disorder rate in 20 cardiomyocytes from each mouse, each point represents an averaged percentile of disordered mitochondria from total 80 to 100 mitochondria in each cardiomyocyte. **(E)** and **(F)** 2D segmentation of outer membrane, inner membrane, and cristae in a healthy and disordered mitochondrion, respectively. **(G)** Analysis of mitochondria disorder by comparison the ratio of entire cristae length to the area of the mitochondrial cross section (L_CRIS_/A_MITO_). Healthy mitochondria like the one shown in Panel E have an averaged ratio around 30 μm^−1^ (open circles in top portion of Panel G). In contrast, disordered mitochondria like the one shown in Panel F have a lower ratio (filled circles in lower portion of Panel G). (For interpretation of the references to color in this figure legend, the reader is referred to the web version of this article.)

We observed similar disorganization in mitochondria and in myofibrillar bundles in heart tissues of both BA.5.2 and BQ.1 infected mice (4 mice in each group, Fig. 5**A and B**). In contrast, the control mice showed normal mitochondrial ultrastructure (Fig. 5**C**). The structural alterations in mitochondria are consistent with findings in murine models of viral myocarditis [33,34]. However, we did not observe any collagen fiber or lipofuscin granule in the mice heart.

Furthermore, we analyzed the mitochondria disorder rate and cristae density ratio in 2D images, Fig. 5**D** exhibits that the mitochondria disordered rates are round 60–75 % in both BA.5.2 and BQ.1 infected mice, whereas no disordered mitochondria were found in the mock group. Fig. 5E–G indicate that cristae density L_CRIS_/A_MITO_ ratio in healthy human mitochondria is around 32 μm^−1^. In contrast, the rates in disordered mitochondria, markedly decreased to 9–15 μm^−1^, indicating a significant loss of cristae ultrastructure in both BA.5.2 and BQ.1 infected cardiomyocytes.

### Proteomic profiling of SARS-CoV-2 infected mouse heart tissues

**Fig. 6:**
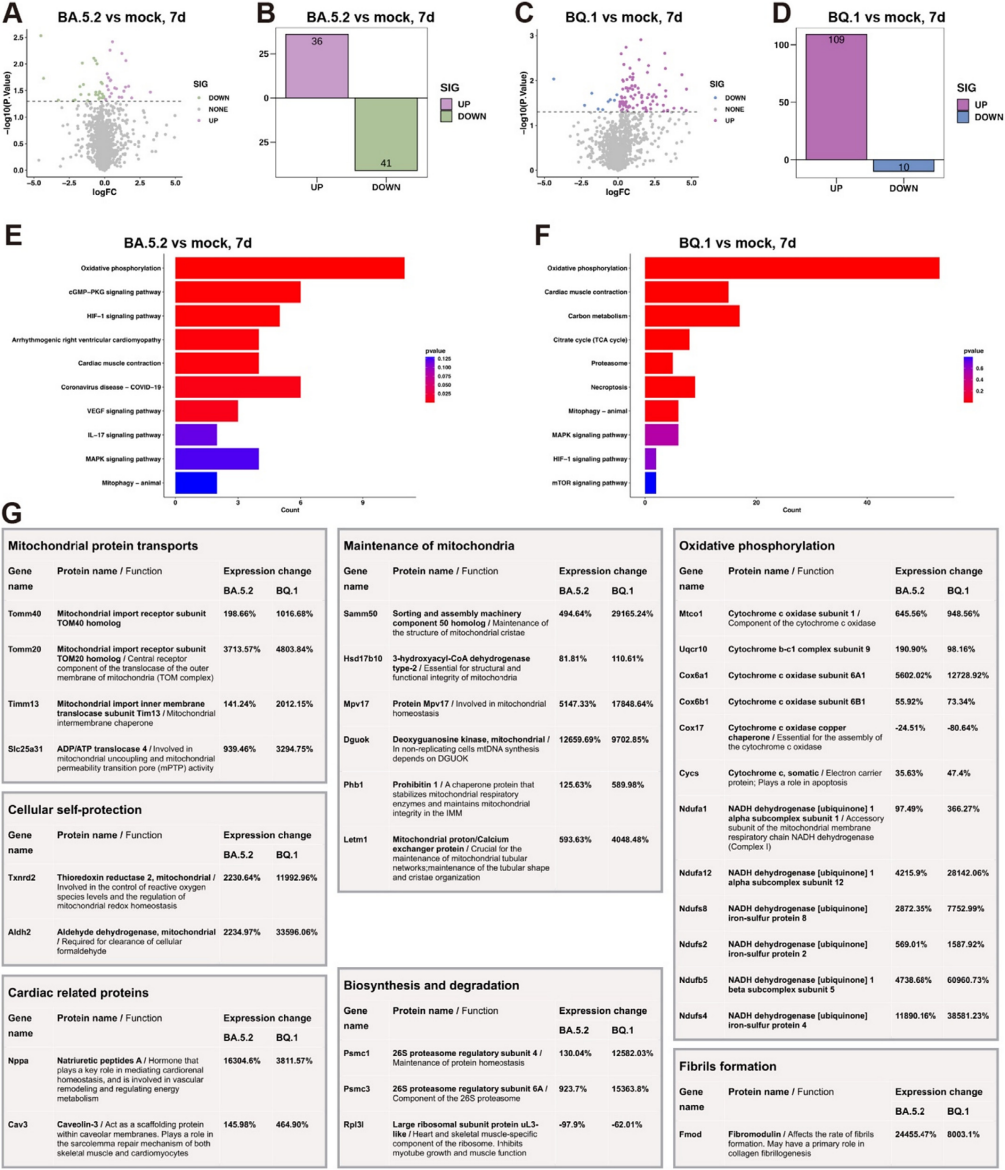
**Proteomic profiling of SARS-CoV-2 infected mouse heart tissues. (A)** and **(C)** Volcano plots representing differentially abundant proteins in BA.5.2 and BQ.1 infected mice heart tissue compared with mock group (n = 4). Green and blue dots represent proteins with a log2 fold change < −0.5 and a q value < 0.05. Plum and purple dots represent proteins with a log2 fold change > 0.5 and a q value < 0.05. Gray dot line indicates q value = 0.05. Only proteins with > 2 identified unique peptides were considered. **(B)** and **(D)** Numeric quantification of colored data points in A and C. **(E)** and **(F)** KEGG pathway enrichment analysis of significantly changed proteins. **(G)** Groups of proteins that upregulated or downregulated in BA.5.2 and BQ.1 infected mice heart, percent changes in protein level are indicated for each protein. (For interpretation of the references to color in this figure legend, the reader is referred to the web version of this article.)

We performed quantitative mass spectrometry analysis of heart tissues from BA.5.2 and BQ.1 infected mice, as well as from a mock group (*n* = 4, Fig. 6). Specifically, when comparing the BA.5.2 group to the mock group, 36 differentially expressed proteins (DEPs) were significantly upregulated, and 41 DEPs were significantly downregulated (Fig. 6**A and B**). When comparing the BQ.1 group to the mock group, 109 DEPs were significantly upregulated, and 10 DEPs were significantly downregulated (Fig. 6**C and D**). To explore the potential functions of these differentially expressed proteins, we conducted Kyoto Encyclopedia of Genes and Genomes (KEGG) pathway enrichment and found functional overlaps between BA.5.2 and BQ.1. The enriched KEGG pathways of DEPs in were involved in oxidative phosphorylation, cardiac muscle contraction, and mitophagy (Fig. 6**E and F**).

Further functional enrichment analysis indicated that the most significantly changed genes and proteins in BA.5.2 and BQ.1 infected heart were related to mitochondria (Fig. 6**G**), including mitochondrial protein transports (mitochondrial import receptors TOM40 and TOM20, mitochondrial import inner membrane translocase TIM13, and Slc25a31 ADP/ATP translocase); maintenance of mitochondria (Sorting and assembly machinery component Samm50, dehydrogenase Hsd17b10, Protein Mpv17, Deoxyguanosine kinase Dgouk, Prohibitin 1, and mitochondria protin/calcium exchanger Letm1); oxidative phosphorylation (cytochrome *c* oxidases, cytochrome bc1 complex, and NADH dehydrogenases); and cellular self-protection (mitochondrial thioredoxin reductase 2 and mitochondrial aldehyde dehydrogenase). Most proteins related to mitochondria are upregulated, likely compensating for protein lost, damage repair, and the generation of new mitochondria.

***Mitochondrial protein transports*** (1). **TOM40:** Most mitochondrial proteins are synthesized as precursors in the cytosol and imported by the translocase of the outer mitochondrial membrane (TOM). The β-barrel protein TOM40 forms the protein-conducting channel of the TOM complex and acts as the central porin of TOM complex to import of precursor proteins into the inter-OMM-IMM space [35]. Consequently, deficits in TOM40 adversely affect preprotein import, disrupting mitochondrial homeostasis and cell viability [36] and arresting animal growth [37]. On the other hand, over-expression of TOM40 is associated with elevated mitochondrial membrane potentials, respiratory rates, cellular ATP levels, suggesting that increased protein level of TOM40 may be protective of mitochondrial function [38]. (2). **TOM20** is an OMM protein that functions as a component of the TOM complex. The primary role of TOM20 is receptor recognition, targeting mitochondrial precursor proteins and guiding them to TOM40 for protein translocation [39]. Tom20 depletion is known to induce a grossly altered morphology of mitochondria, Tom20-deficient fungi contain mitochondria that are highly deficient in cristae ultrastructure [40]. The lack of cristae in TOM20-deficient mitochondria is similar to what we observed in human and mouse cardiomyocytes post SARS-CoV-2 infection. It remains unclear if SARS-CoV-2 affects TOM40 and TOM20, nevertheless, a recent study revealed that the ORF9b protein of both SARS-CoV and SARS-CoV-2 physically interacts with TOM70, a partner of TOM20 that also recognizes and transfers mitochondrial preproteins to TOM40, and that the expression level of TOM70 was found decreased during SARS-CoV-2 infection [41]. (3). **TIM13** is a member of translocase of the inner mitochondrial membrane (TIM) family of chaperones, TIM13 functions in the inter-OMM-IMM space to shield unfolded, hydrophobic membrane proteins and maintain an import-competent state for downstream translocation [42,43]. Additionally, a recent study demonstrated that TIM13 is closely related to fibrosis in liver disease, silencing TIM13 significantly reduced the expression of profibrogenic genes and apoptosis related genes, although the underlying mechanism has yet to be fully elucidated [44]. (4). **Slc25a31** is a member of the ADP/ATP carrier family of proteins that exchange cytosolic ADP for matrix ATP in the mitochondria. Over-expressing of Slc25a31 has been shown to display an anti-apoptotic phenotype [45].

***Maintenance of mitochondria*** (1). **Samm50** is the core component of the mitochondria sorting and assembly machinery (SAM), integral to the maintenance of mitochondrial structure and function. Recent studies have further elucidated its roles in mitochondrial dynamics, morphology, and bioenergetics. Samm50 plays a key role in the OMM, and it has been confirmed that Samm50 promotes the biogenesis of TOM40 by forming a SAM-TOM super-complex, and Samm50 deficiency inhibits the assemble of TOM40 [46,47]. This disruption can lead to impaired mitochondrial biogenesis and maintenance, resulting in decreased mitochondrial function and altered energy metabolism. Studies have shown that reduced expression of Samm50 leads to the development of myocardial hypertrophy and associated mitochondrial dysfunctions, including impaired mitophagy and increased oxidative stress in mouse ventricular cardiomyocytes [48]. In human and murine myotubes, Samm50 deficiency leads to significant mitochondrial fragmentation, characterized by reduced volume, surface area, and complexity [49]. Additionally, Samm50 interacts with key proteins involved in mitochondrial dynamics, such as Drp1, a GTPase responsible for mitochondrial fission. Overexpression of Samm50 in HeLa cells induces Drp1-dependent mitochondrial fragmentation. Conversely, Samm50 knockdown results in swollen mitochondria with impaired cristae structure, reduced levels of OPA1 (a protein crucial for inner membrane fusion), and compromised mitochondrial inheritance [50]. Moreover, Samm50 physically interacts with Mitofilin and CHCHD6, two core proteins of the mitochondrial contact site and cristae organizing system (MICOS) complex in the junction of IMM and cristae, Samm50 is indispensable for cristae structure stability and the proper assembly of the mitochondrial respiratory chain complexes [51]. Depletion of Samm50 leads to changes in mitochondrial shape and the loss of mitochondrial cristae [52,53]. Collectively, these findings underscore Samm50′s multifaceted role in preserving mitochondrial integrity, regulating dynamics, and ensuring optimal mitochondrial function across various cell types and physiological contexts. (2). **Hsd17b10** encodes 3-hydroxyacyl-CoA dehydrogenase type-2, a member of the short chain dehydrogenase/reductase superfamily. This mitochondrial protein is involved in pathways of fatty acid, branched chain amino acid and steroid metabolism and has been reported to be associated with mitochondrial toxicity in neurodegenerative diseases [54]. Loss of Hsd17b10 function mediated by gene mutation, knock-down, or knock-out causes mitochondrial dysfunction and apoptotic cell death. Moreover, EM analysis of mitochondria from *Xenopus* embryos, mouse brain, and human fibroblasts showed that Hsd17b10 protein is required for structural and functional integrity of mitochondria [55]. (3). **MPV17** is an IMM protein and its deficiency can cause mitochondrial DNA (mtDNA) depletion, increase reactive oxygen species (ROS), and promote mitochondrial apoptosis [56,57]. Moreover, Electron microscopy examination observed broken or disappearance of cristae in MPV17 knock-out zebrafish and mice, and in Sym1 (yeast ortholog of MPV17) ablation yeast [[58], [59], [60]]. (4). ***DGUOK*** gene encodes mitochondrial deoxyguanosine kinase, which mediates phosphorylation of purine deoxyribonucleosides in the matrix. DGUOK is crucial for the maintenance and replication of mtDNA [61]. DGUOK deficiency leads to mtDNA depletion syndrome (MDS), which results in reduced mtDNA content and decreased activity of the mtDNA-encoded respiratory chain complexes I, III, IV, and V [62]. The depletion impairs the mitochondrial respiratory chain function, leading to decreased ATP production and increased production of ROS. The resulting oxidative stress further damages mitochondrial components, exacerbating the dysfunction. DGUOK-related MDS is characterized by structural abnormalities in mitochondria, such as loss of cristae integrity and swollen mitochondria [63,64]. (5). **Prohibitin 1 (PHB1)** is a highly conserved protein localized in the IMM, playing a crucial role in maintaining mitochondrial structure and function. PHB1 with its homolog PHB2, forms a large oligomeric ring structure in the IMM, serving as scaffolds that stabilize mitochondrial proteins and lipids [65]. This structural organization is vital for preserving cristae morphology and ensuring the proper assembly of respiratory chain complexes [66]. Prohibitins function as chaperone proteins that stabilizes mitochondrial respiratory enzymes and maintains mitochondrial integrity in the IMM and cristae [66]. Prohibitins modulate mitochondrial dynamics by interacting with key proteins involved in fusion and fission processes. It influences the processing of OPA1, a dynamin-like GTPase essential for inner membrane fusion, thereby affecting mitochondrial morphology and function [67]. When Prohibitins are deficient, there is a disruption in these dynamics, leading to impaired mitochondrial division and fusion, affecting the mitochondrial membrane potential and results in an aberrant cristae morphogenesis [67]. PHB1 plays a protective role against oxidative stress, over-expression of PHB1 in cardiomyocytes has been shown to protect cells from oxidative stress-induced mitochondrial membrane permeability, inhibited release of cytochrome *c*, and suppressed mitochondrial apoptosis [68], treatment with recombinant human PHB1 enhances the antioxidant/anti-inflammatory response and protects HL-1 cardiomyocytes [69]. Additionally, PHB1 plays a crucial role in mitophagy, the selective degradation of damaged mitochondria, it facilitates the recruitment of mitophagy receptors Nix/Bnip3L to mitochondria, particularly in response to elevated ROS levels. This helps to clear out dysfunctional mitochondria, maintaining cellular homeostasis [70]. Moreover, mitochondrial shape and ultrastructure are affected by the lipid composition of mitochondria. Prohibitins affect the maturation of cardiolipin, a phospholipid of the mitochondrial inner membrane that plays a role in mitochondrial fusion [71]. (6). **Letm1** is a mitochondria protin/calcium exchanger localized to the IMM [72]. Letm1 maintains the mitochondrial tubular shapes and is required for normal mitochondrial morphology and cellular viability, LETM1 downregulation cause mitochondrial swelling and cristae disorganization, formation of the respiratory chain complexes are also impaired by LETM1 knockdown [73].

***Oxidative phosphorylation***. Multiple genes and proteins involve in **cytochrome *c* oxidase**, **cytochrome bc1 complex**, and **NADH dehydrogenas**. NADH dehydrogenas known as Complex I, Cytochrome bc1 complex as Complex III, and cytochrome *c* oxidase as Complex IV of the mitochondrial electron transport chain. They are the enzymes of the mitochondria electron transport chain and play crucial roles in cellular energy production, the process is essential for maintaining the proton gradient that drives ATP synthesis. Impairment of NADH dehydrogenas, cytochrome bc1 complex, and cytochrome *c* oxidase activities disrupt the electron transport chain, leading to reduced ATP production, increased ROS production, and impaired cellular respiration. These changes result in various structural and functional abnormalities in mitochondria, including altered mitochondrial morphology, increased mitochondrial fragmentation, and swelling [74].

***Cellular self-protection*** (1). Gene ***Txnrd2*** encodes thioredoxin reductase 2, a selenocysteine-containing enzyme essential for mitochondrial oxygen radical scavenging. Thioredoxin reductase 2 is crucial for maintaining redox homeostasis within mitochondria by reducing oxidative stress [75]. Cardiac-specific deletion of Txnrd2 in mice results in dilated cardiomyopathy, the lack of Txnrd2 results in increased oxidative damage, disrupted mitochondrial dynamics, and impaired energy production, leading to structural alterations such as mitochondrial fragmentation and loss of cristae integrity [76]. (2). Gene ***Aldh2*** encodes aldehyde dehydrogenase 2, an enzyme predominantly found in the mitochondrial matrix, crucial for detoxifying aldehydes generated during oxidative stress. ALDH2 deficiency leads to increased accumulation of toxic aldehydes, which impaired mitochondrial respiration, reduced ATP production, and increased ROS generation, leading to oxidative damage to mitochondrial DNA, proteins, and lipids [77]. The lack of ALDH2 exacerbates mitochondrial structural abnormalities, including swelling and loss of cristae integrity [78].

Other proteins involved in fibrils formation (fibromodulin), biosynthesis and degradation (26S proteasomal subunits), as well as cardiac related proteins (natriuretic peptides A and caveolin-3), were upregulated in BA.5.2 and BQ.1 infected mice heart. One ribosomal subunit protein, Rpl3l, which inhibits myotube growth and muscle function in skeletal and cardiac muscle [79], was down-regulated.

Interestingly, no SARS-CoV-2 proteins were detected in the cardiac proteome at 7 dpi, which aligns with the known viral kinetics, tissue tropism, and the limitations of the BALB/c mouse model for studying direct cardiac infection by SARS-CoV-2. In our study, BALB/c mice were intranasally infected with either BA.5.2 or BQ.1, both of which are known to establish a mild infection in this animal model [80]. These mice do not exhibit body weight loss, but viral titers and histopathological changes can be detected in the lungs during the early phase of infection. However, SARS-CoV-2 is rapidly cleared in BALB/c mice, with live virus typically undetectable in the lungs by day 5 post-infection. Therefore, by day 7, the animals are in a recovery phase with minimal to no detectable viral replication [81,82]. It is important to emphasize that SARS-CoV-2 primarily targets the upper respiratory tract and lung tissues [83], direct viral replication in cardiac tissue remains controversial and has not been consistently demonstrated, particularly in wild-type mice such as BALB/c [[84], [85], [86], [87]]. While the BA.5 lineage has demonstrated increased affinity for human ACE2, this affinity remains insufficient to mediate effective infection of BALB/c mouse cardiomyocytes [88]. Given the limitations of viral tropism in this model, it is unsurprising that our proteomic analysis of cardiac tissue at day 7 dpi did not identify any SARS-CoV-2-derived proteins.

## Discussion

The global COVID-19 pandemic has presented clinicians with the daunting task of diagnosing and treating patients with non-specific cardiovascular symptoms, complicated by ambiguous CMR findings. Myocarditis, characterized as an inflammatory disease of the myocardium, requires immunological and histological confirmation for diagnosis, and endomyocardial biopsy serves to gain certainty about the diagnosis and identify the potential cause of the disease. Despite Patient #1 lacking typical viral myocarditis symptoms such as chest pain and dyspnea, elevated cardiac troponin levels prompted further investigation. Following the “2022 Expert Consensus Decision Pathway on Cardiovascular Sequelae of COVID-19 in Adults: Myocarditis and Other Myocardial Involvement, Post-Acute Sequelae of SARS-CoV-2 Infection, and Return to Play” by the American College of Cardiology [89], we proceeded with endomyocardial biopsy, aligning with international expert consensus that published in 2021 by the Heart Failure Association of the European Society of Cardiology, the Heart Failure Society of America, and the Japanese Heart Failure Society, in which endomyocardial biopsy is recommended in diagnostic assessment of select patients with atypical myocarditis [90]. In this study, immunohistochemical analysis, Masson's trichrome staining, HE staining, and electron microscopy exam were performed on the endomyocardial biopsy samples, providing precise evidence for the diagnosis of myocarditis. We encourage clinicians to consider endomyocardial biopsy following the above expert consensuses, particularly in diagnosis of Long COVID associated cardiovascular outcomes.

SARS-CoV-2 primarily targets the respiratory tract, especially the upper airways and lung parenchyma [83]. Although clinical and autopsy studies have shown that COVID-19 may cause myocardial inflammation or injury, accumulating evidence suggests that such effects are largely secondary, mediated through systemic immune responses, inflammatory cytokines, or microvascular dysfunction, rather than direct viral replication in cardiomyocytes [[84], [85], [86], [87]]. A recent molecular crosstalk analysis has integrated and analyzed two Gene Expression Omnibus (GEO) datasets (SARS-CoV-2 infected human induced pluripotent stem cell-derived cardiomyocytes and heart failure samples), common differentially expressed genes of both datasets were identified, functional enrichment analysis showed that mitochondrial dysfunction, metabolic abnormality, immune activation, and inflammatory response were the most important features of COVID-19 and cardiovascular diseases. This comprehensive molecular crosstalk analysis has identified immune-inflammation and mitochondrial metabolism-related genes were key genes that connecting COVID-19 and cardiovascular diseases [91].

The evidence for active viral replication in the heart, particularly in non-transgenic wild-type mice such as BALB/c, remains limited and inconclusive. Notably, the BALB/c mouse is a non-permissive model for SARS-CoV-2 infection due to its lack of human ACE2 expression. Although the BA.5 variant has improved binding affinity for hACE2, this does not translate into efficient infection of murine cardiomyocytes [88]. Therefore, any cardiac abnormalities observed in this model likely reflect systemic or indirect responses rather than direct viral cytopathic effects.

Moreover, the BALB/c mouse model offers a high degree of experimental reproducibility. This consistency arises from several controlled factors: the mice are genetically homogeneous, maintained under standardized housing conditions, and infected with the same viral strain at identical doses and via the same route. These variables—strain background, genotype, infection dose, and environment—are tightly regulated, allowing us to isolate the biological effects of SARS-CoV-2 infection with minimal confounding, and to generate highly reproducible phenotypic outcomes across individuals. By contrast, clinical patients inevitably present a high degree of heterogeneity. The cohort in our study spans a wide age range and includes individuals with varying comorbidities, immune backgrounds, prior exposures, and treatment histories. Moreover, the onset and progression of myocardial injury in COVID-19 are influenced by a complex interplay of viral factors, host immune responses, and pre-existing cardiovascular conditions. As such, variability in clinical presentation and tissue pathology is expected and reflects the real-world complexity of human disease.

The presence of lipofuscin granules in cardiomyocytes, indicative of cellular damage and aging, has been historically associated with myocardial hypertrophy, heart failure, and sudden cardiac death [21,92]. These granules result from the oxidation and polymerization of protein and lipid residues, often accumulating when mitochondria suffer structural damage, underscoring their significance as markers of cellular distress [93]. When mitochondria undergo structural damage, lysosomes, which are normally responsible for mitochondrial turnover, accumulate lipofuscin granules [94].

We have observed an interesting phenomenon in the mitochondria, the swollen and vacuolated mitochondria, distorted and broken cristae, all indicated severe damage to this important cellular organelle. This damage is highly likely caused by the SARS-CoV-2 Infection, as we have found an almost identical mitochondria disorganization in the mice that were infected with SARS-CoV-2. Our findings are consistent with a recent study in which mitochondrial gene expression was analyzed in autopsy tissues from patients with COVID-19, and core mitochondrial gene expression were suppressed in the hearts, chronically impaired mitochondrial function [95].

Mitochondrial damage has profound implications for cellular respiration, ATP production, and metabolism, potentially precipitating cardiovascular, neurodegenerative, and metabolic disorders. Notably, our preliminary observations, derived from a limited patient sample, suggest persistent mitochondrial damage in cardiomyocytes up to three months post-COVID-19 recovery, challenging existing recommendations on exercise cessation post-infection. While current guidance from the ACC’s Sports and Exercise Cardiology Council recommends a two-week cessation of exercise for mildly or moderately symptomatic COVID-19 athletes [96], our findings—along with prior guidance advocating for 3–6 months of rest for individuals with clinical myocarditis [97]—suggest that these recommendations warrant further investigation in future studies. Moreover, our findings support previous studies and reviews, patients report remitting multi-system symptoms for weeks and months with some failing to recover after 2 years [98,99], and the Long COVID symptoms shift from respiratory to renal, musculoskeletal cardiovascular, and neurological disorder [9,[100], [101], [102]].

Our current study does not include functional assessments such as ATP production or ROS levels in the cardiomyocytes, and that such assays would undoubtedly provide deeper mechanistic insights. Moreover, given the small sample size (n = 5), our findings are exploratory and require validation in larger, prospective studies to assess the generalizability and long-term cardiovascular consequences of COVID-19. These preliminary results highlight a potential need to consider extended recovery periods before resuming exercise following COVID-19, though this hypothesis must be confirmed in larger cohorts.

## Clinical study

All Clinical studies were approved by the Human Ethics Committee of Shanghai Tenth People’s Hospital, Tongji University School of Medicine (Approval Number: 23KT45). Written informed consent was obtained from the patient. The studies were performed in accordance with the Declaration of Helsinki.

## Animal experiments

All animal studies were performed following guidelines from the Directive 2010/63/EU of the European Parliament on the protection of animals used for scientific purposes. All protocols were approved by the Institutional Animal Care and Use Committee (IACUC) of The First Affiliated Hospital of Guangzhou Medical University (Approval Number: 20230451).

## CRediT authorship contribution statement

**Wenliang Che:** Conceptualization, Methodology, Investigation, Resources, Writing – original draft, Writing – review & editing, Supervision, Funding acquisition. **Shuai Guo:** Data curation, Investigation, Writing – original draft. **Yanqun Wang:** Investigation, Data curation, Resources, Funding acquisition, Writing – original draft, Writing – review & editing. **Xiaohua Wan:** Software, Investigation, Data curation, Resources, Funding acquisition. **Bingyu Tan:** Investigation. **Hailing Li:** Investigation. **Jiasuer Alifu:** Investigation. **Mengyun Zhu:** Investigation. **Zesong Chen:** Investigation. **Peiyao Li:** Investigation. **Lei Zhang:** Investigation. **Zhaoyong Zhang:** Investigation. **Yiliang Wang:** Investigation. **Xiaohan Huang:** Investigation. **Xinsheng Wang:** Software, Investigation, Data curation. **Jian Zhu:** Investigation. **Xijiang Pan:** Investigation, Resources. **Fa Zhang:** Software, Investigation, Resources, Funding acquisition. **Peiyi Wang:** Investigation, Resources, Funding acquisition. **Sen-Fang Sui:** Resources, Validation, Writing – review & editing. **Jincun Zhao:** Methodology, Resources, Supervision, Funding acquisition. **Yawei Xu:** Conceptualization, Methodology, Resources, Writing – original draft, Supervision, Funding acquisition. **Zheng Liu:** Conceptualization, Methodology, Investigation, Validation, Resources, Writing – original draft, Writing – review & editing, Supervision, Funding acquisition.

## Declaration of competing interest

The authors declare that they have no known competing financial interests or personal relationships that could have appeared to influence the work reported in this paper.
